# News and Notes

**Published:** 1900-05

**Authors:** 


### *NEWS AND NOTES.*

The *Stylus* is a new publication emanating from St. Louis with its first issue. St. Louis never does things by halves.

The S. S. White Dental Manufacturing Co. have moved to handsome and very extensive quarters in the Prudential Building.

Dr. S. S. Pittman died at his home in Chipley, Ga., on April 22d. Dr. Pittman was one of the most prominent physicians in his section.

Dr. J. H. Spier of Cartersville, Ga., died in that city on April 23d. On account of his health, Dr. Spier had not been in active practice for several years.

Dr. J. W. Jones, Lelie, Ga., died in Bartow, Fla., on March 24th, from an overdose of chloroform.

Dr. Norman B. Gwyn, University of Toronto, '96, Assistant Resident Physician of Johns Hopkins Hospital, has resigned to accept a position in Philadelphia. The vacancy has been filled by the appointment of Dr. Gordon Wilson, University of Virginia, '99.

According to the press dispatches, Congressman Boutelle of Maine, who has been in a sanatorium for several months, was renominated on the statement of his physician that such action might effect a cure, whereas failure to secure a renomination would probably kill him.

A general order has been issued by the War Department governing the employment of special nurses for sick officers and soldiers at stations where treatment in an army hospital cannot be obtained. In such cases it is stipulated that the attending physician shall certify that the services of the nurse were indispensable. The rate of compensation is the same as that allowed army nurses serving in the United States, namely, $10 per week.

---

A FEATURE of the reunion of Confederate veterans, to be held at Louisville, Ky., on May 30th, will be the meeting of the Confederate Surgeons' Association. A Medical Committee has been appointed by the General Committee, and Dr. Preston B. Scott, one of the two surviving medical directors of the Confederate Army, has been made chairman. It is stated that the whereabouts of about 1,500 ex-Confederate surgeons is known to the committee, which will use every effort to secure a large attendance.

---

IT is understood that a board of medical officers convened to investigate the merits of the Woodbridge treatment of typhoid fever, as carried out at the Fort Myer General Hospital during the war with Spain by Dr. Woodbridge himself, then Major and Surgeon United States Volunteers, finds a mortality of about ten per cent. of all cases treated by the Woodbridge method, and about seven per cent. of all cases treated by other methods. In all, about 600 cases of typhoid fever were treated at the Fort Myer Hospital; of these, 57 were treated by Dr. Woodbridge, who was afforded every facility in the application of his treatment.

---

WE clip the following from a daily paper: "A bacteriologist asked a woman who did not usually have to go on very dirty streets, if he might make an experiment on one of her skirts. It was a comparatively new one, and of course received the daily brushing too. He found on part of the skirt-binding at the hem the following small menagerie: Two hundred thousand germs, many bearing diphtheria, pneumonia, and tonsillitis; also collections of typhoid and consumption microbes. The owner has been converted to the short skirt." We would ask how the germs were counted, and also in what way they are supposed to "bear" diphtheria, pneumonia and tonsillitis? There is some danger of overdoing the germ theory.

---

RECENTLY a pregnant woman in the St. Luke's Hospital, Chicago, shortly before her confinement, while being transferred from one floor to another, had her leg caught in the elevator and crushed. When the child was born his left foot, left side and left leg were paralyzed and deformed. The mother brought suit against the hospital to recover damages to the extent of $50,000 for the injuries sustained by the child before his birth. A general demurrer was sustained by the Supreme Court of the State of Illinois on the ground that at the time of the accident the child could not be credited as a separate being capable of sustaining an action independent of the mother. "If the action can be maintained," the court says, "it necessarily follows that an infant may maintain an action against its own mother for prenatal injuries."

---

THE proposition having been recently advanced that, in view of the dangers of appendicular inflammation and the comparative ease and innocuousness of the operation in uncomplicated cases, its removal in the case of all young children should be practiced as a routine measure, as Jews practice circumcision, the editor of the *Medical Review* invited a number of representative American surgeons to express their views thereon. The surgeons' answers are published in the *Review* for March 17th, and the result is almost exactly what we should have expected—viz., that out of eighty-eight surgeons replying to the query, eighty-three express themselves more or less strongly in condemnation of the proposition. We say "almost exactly" what we should have expected advisedly. Our only surprise is that five should have been found to write with toleration of it.—*N. Y. Medical Journal.*

